# The Association Between Impaired Awareness and Depression, Anxiety, and Apathy in Mild to Moderate Alzheimer's Disease: A Systematic Review

**DOI:** 10.3389/fpsyt.2021.633081

**Published:** 2021-02-04

**Authors:** Ignacia Azocar, Gill Livingston, Jonathan Huntley

**Affiliations:** Division of Psychiatry, University College London, London, United Kingdom

**Keywords:** Alzheimer's disease, awareness, depression, anxiety, apathy

## Abstract

**Objectives:** Impaired awareness of cognitive and functional deficits is a common feature of Alzheimer's disease (AD). Although a lack of awareness has been suggested to be a protective factor against experiencing affective symptoms, such as depression, anxiety, and apathy which are common in AD, there is conflicting evidence about the links between them. This systematic review examines the evidence for an association between impaired awareness and depressive, anxiety, and apathy symptoms in mild to moderate AD.

**Method:** We searched four databases (OvidMedline, Embase, PsycInfo, and PsycArticles) using terms encompassing awareness, apathy, depression, anxiety, and mild-moderate AD. We included studies that assessed the relationship between awareness and depressive symptoms, anxiety symptoms, or apathy. We assessed included papers for quality and report results using a narrative approach, prioritizing high quality studies.

**Results:** We identified 1,544 articles, and twenty-seven studies fulfilled inclusion criteria (high-quality = 15; moderate-quality = 12). Most high-quality studies reported that impaired awareness in early-stage AD is cross-sectionally linked with fewer depressive symptoms and anxiety symptoms (correlation ranged from −0.3 to −0.7), but with more apathy.

**Conclusions:** High-quality studies suggested that in people with early AD, impaired awareness is related to fewer depressive and anxiety symptoms and to more apathy. Future research should focus on elucidating causality among impaired awareness and these symptoms in AD.

## Introduction

Dementia is characterized by progressive cognitive decline, the presence of neuropsychiatric symptoms (NPS) and difficulties in activities of daily living (ADL) (1). The worldwide prevalence of dementia is currently around 50 million (2). Alzheimer's disease (AD) is the leading cause of dementia, corresponding to 62% of all dementia cases (3), and people with AD may lack insight or awareness of the illness or symptoms. Awareness is a construct related to accurate self-appraisal, and it has been defined as having a “realistic perception of one's personal situation, performance and functioning” (4). Awareness is not a unitary concept, and should be considered in relation to the specific objects or domains of awareness being assessed. In AD, people may have impaired awareness of different domains, such as of cognitive impairment, of deficits in ADLs, of affective symptoms or behavioral changes (5); and, a partial or complete absence of awareness of having an illness (6). Impaired awareness of deficits is a marked and common clinical feature of AD (7). Sixty percent of people with mild dementia present some degree of impaired awareness, and the prevalence and frequency increases with the progression of the disease (8). Impaired awareness of illness or deficits in AD, has also been referred to as *lack of insight* or *anosognosia* (9) and constitutes a challenge to treatment adherence, decreases quality of life, is associated with a worse prognosis, and increases carer and family burden (6, 10). For the purpose of this review, the term awareness is used to refer to awareness of illness, or of cognitive, functional or behavioral change related to AD.

Three common approaches are used to assess impaired awareness in AD: (1) clinician ratings of patient's awareness of illness; (2) the discrepancy between prediction of performance and actual performance; and (3) the discrepancy between patient and caregiver scores on cognitive, functional or behavioral outcomes (11). The first approach may be part of a routine clinical assessment, or involve a semi structured interview, where clinicians make a judgement about impaired awareness of illness or in a specific domain (12). A disadvantage of this approach is that it relies solely on the judgement of the clinician, and often considers impaired awareness as a unitary symptom that can be categorized by the clinician into, for example, “no,” “partial,” or “full” awareness of deficits (12). The second strategy scores the degree of awareness as the difference between an estimation of performance predicted by the patient on a neuropsychological task and the actual score achieved (7). This has the advantage of assesing symptomatology from the patient perspective, and providing a direct comparison of subjective and objective neuropsychological function, however is mainly limited to assessing awareness of cognitive performance (11). Moreover, there is the potential of bias, as a patient may over- or underestimate their performance for a variety of reasons which may be culturally orcontextually mediated, rather than related to dementia. The third strategy compares ratings given by patients regarding their performance on changes of cognition, mood, behavior, or ADLs against caregivers rating (13). The main limitation of this approach is that it assumes a caregiver is providing an objectively accurate rating, whereas, there is the potential for bias due to caregiver burden and distress (11). Nonetheless, significant correlations between caregiver and clinician reports have been found, with reports given by carers found to be accurate and reliable (5, 14). The extent of awareness varies across people at each stage of AD, and relationships regarding impaired awareness and NPS in mild to moderate AD remain uncertain (15, 16). In AD, depression and apathy are relatively common NPS, with a pooled prevalence of 42 and 49%, respectively (17). Anxiety is also a distressing affective symptom that affects 39% of the population living with AD (17). It remains unclear whether intact awareness of illness, health-loss and impairments in early AD is linked with these symptoms of depression, anxiety, and apathy; or if, on the other hand, these symptoms occurs independently of the level of awareness and insight, as a consequence of the neurodegenerative process or other environmental and individual variables. There is conflicting evidence particularly with depression, where some studies report that people with greater awareness of disease suffer from more depressive symptoms, potentially as an emotional and psychological reaction to having the illness (18, 19). Although the mechanisms underlying the relationship between awareness of disease and depression remain unclear (20), and other studies have reported no association between level of awareness and the presence of depressive symptoms (12, 13, 21). Less conflicting evidence has been found for apathy, where correlations between impaired awareness and more apathetic symptomatology have been previously reported (22, 23), although these studies included participants with severe AD. The association between anxiety and awareness remains unclear, as anxiety is often assessed only as a secondary outcome, however, one review has reported that unawareness is associated with lower anxiety scores in AD (11).

This systematic review aims to describe the associations between impaired awareness and the presence of depressive, anxiety, and apathy symptoms in early-AD; and in light of the results, consider possible causalities and underlying mechanisms of this relationship. Elucidating links between impaired awareness and these symptoms in early-AD, would provide a more in-depth understanding of the characteristics and progression of the disease, as impaired awareness, affective symptoms, and apathy are all related to prognosis of the disease, quality of life, and carer burden (15).

Understanding the role of awareness as a possible protective or risk factor for affective and apathetic symptoms, may aid in delivering more targeted psychological therapy in early AD, including awareness as a variable to consider when deciding therapeutic approaches. Thus, clarifying the relationship between awareness, and common symptoms in early AD, such as depression, anxiety, and apathy, could inform treatment and management plans, and may have a positive impact both for the person with AD and for their carers and family.

## Methods

### Search Strategy

The search strategy was developed according to PRISMA guidelines (24). We searched four databases (Embase, PsycInfo, OvidMedline, and PsycArticles) from inception to 27th of October 2020. We used the following search terms, with the Boolean operators “AND” and “OR” used to combine terms and concepts: “Alzheimer's disease” or “AD” or “Alz^*^” or “dementia” or DAT, AND “metacognition” or “aware^*^” or “metacognitive” or “insight” or “anosognosia” or “self-appraisal,” AND “depress^*^” or “low mood” or “major depressive episode” or “MDE” or “anxiety” or “apathy” or “neuropsychiatric symptoms” or “BPSD.”

#### Selection Criteria

Inclusion criteria were published, peer reviewed, original research studies of people with a diagnosis of mild-to-moderate AD. A diagnosis of AD was required to have been made using validated criteria, such as the National Institute of Neurological Disorders and Stroke–Alzheimer Disease and Related Disorders (NINCDS–ADRDA) or the Diagnostical Manual of Mental Disorders (DSM); and validated methods used to measure awareness and depression, anxiety, or apathy were required. We included studies in English or Spanish. As we only included data related to mild and moderate AD, we excluded studies containing only participants with other non-AD dementias, mild cognitive impairment, or severe dementia. Gray literature and unpublished studies were excluded.

#### Eligibility of the Studies and Quality Assessment

All potential studies were screened by two authors for eligibility and disagreements were discussed with a third independent reviewer.

We extracted relevant data from the included studies including study authors, year of publication, study design, number of participants, severity of AD, neuropsychiatric measure used, awareness domain and assessment measure, statistical method and association between awareness and neuropsychiatric symptom, and study conclusions.

For quality assessment we used the Newcastle-Ottawa instrument for case-control and cohort-studies (NOS) (25) and the NOS adapted-version for cross-sectional studies (26) for each study (Appendix 1 in Supplementary Material). There is no well-established threshold to define quality-level, therefore we used the definitions of level of quality implemented from a previous study (26). Total scores out of 10, from 1 to 4 were defined as low-quality, from 5 to 7 as moderate-quality, and equal or higher to 8 as high-quality.

## Results

### Search Results

**Figure 1:**
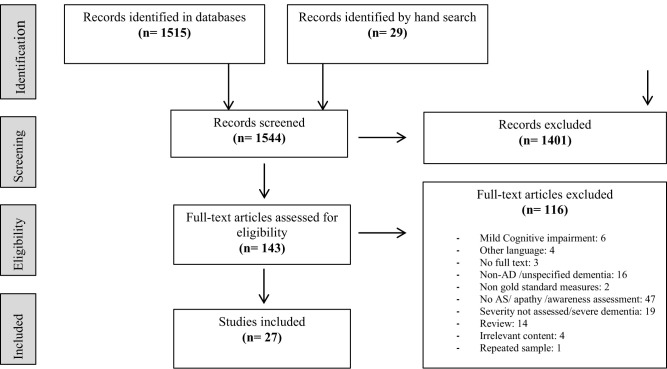
PRISMA flow chart.

We identified 1,544 references in the literature searches. A PRISMA flow chart of the search is shown in Figure 1. After removal of duplicates and irrelevant articles, 143 articles were selected for full-text eligibility assessment. Finally, 27 studies fulfilled inclusion and exclusion criteria.

### Study Characteristics

The main characteristics of each individual study are summarized in Table 1.

**Table 1:** Characteristics of included studies.

**Study, country**	**Study design**	**N Severity of AD**	**Awareness concept; Measure**	**NPS assessed**	**NPS measure**	**Quality category (score)**
1. Amanzio et al. (27), Italy	Cross-sectional	117 Mild	Awareness; AQ-D	Apathy	HAM-D MAS	High (9)
2. Bertrand et al. (28), UK	Cross-sectional	20 Mild-Mod	Awareness; AQ-D	Depression, Apathy	GDS AES-C	High (8)
3. Chen et al. (29), Taiwan	Cross-sectional	55 Mild-Mod	Awareness; GRAD (adapted)	Depression, Apathy	CSDD AES-I for Apathy NPI	High (9)
4. Cines et al. (30), USA	Cross-sectional	104 Mild-Mod	Awareness; ARS (modified)	Depression	GDSd	High (9)
5. Clare et al. (31), UK	Prospective	12 Mild -Mod	Awareness; MARS	Depression	HADS BPC	High (8)
6. Clare et al. (4), UK	Cross-sectional	101 Mild-Mod	Awareness; Multidimentio-nal approach: Explicit and implicit awareness^*^	Anxiety	HADS	High (9)
7. Conde-Sala et al. (32), Spain	Cross-sectional	164 Mild-Mod	Anosognosia; AQ-D	Depression, Apathy	GDS NPI	High (8)
8. Conde-Sala et al. (33), Spain	Cross-sectional	141 Mild-Mod	Anosognosia; AQ-D	Depression	GDSd NPI	High (9)
9. DeBettignies et al. (34), USA	Cross-sectional	12 Mild-Mod	Insight; P-C discrepancy on the IADL and PSMS	Depression	Hamilton Depression Scale	Moderate (6)
10. Derousne et al. (35), France	Retrospective	88 Mild-Mod	Awareness; P-C discrepancy on the CDS-IU; Clinician assessment; PBQ	Anxiety, Apathy	By the informant using the PBQ; by the patient using the ZSRSD.	Moderate (6)
11. Gilleen et al. (36), UK	Cross-sectional	27 Mild-Mod	Awareness; SAI-E; UMDS- MD item 1; MARS; aPCRS; DEX.	Depression	BDI-II	Moderate (7)
12. Horning et al. (37), USA	Cross-sectional	107 Mild-Mod	Insight; NRS: item of impaired insight	Depression, Anxiety	NRS AES	Moderate (7)
13. Jacus (38), France	Cross-sectional	20 Mild 20 MCI	Awareness; PCRS, SCSAD	Depression, Apathy, Anxiety	BDI-II AES STAI-T	High (8)
14. Kashiwa et al. (39) Japan	Cross-sectional	84 Mild-Mod	Anosognosia; Squire & Zouzouris Anosognosia Scale (adapted)	Depression	GDSd NPI	High (8)
15. Lacerda et al. (16), Brazil	Cross-sectional	89 Mild-Mod	Awareness; ASPIDD	Depression	CSDD	High (9)
16. Lehrner et al. (40), Austria	Cross-sectional	43 Mild	Awareness; Subtracting VSRT Delayed Recall scores from FAI scores.	Depression	BDI-II	Moderate (7)
17. Mak et al. (41), Singapore	Cross-sectional	36 Mild-Mod	Anosognosia; AQ-D	Apathy, Depression	AES GDS	Moderate (7)
18. Nakaaki et al. (42), Japan	Cross-sectional	42 Mild-Mod	Insight; P-C discrepancy on the short memory questionnaire	Depression	HRSD CES-D NPI	Moderate (6)
19. Oba et al. (43), Japan	Cross-sectional, retrospective	118 Moderate	Anosognosia; Squire & Zouzouris Anosognosia Scale	Depression	GDS	High (8)
20. Smith et al. (44), USA	Longitudinal	23 Mild-Mod	Anosognosia; AII	Depression	GDSd	Moderate (7)
21. Sousa et al. (45), Brazil	Longitudinal	69 Mild	Awareness; ASPIDD	Depression	CSDD	High (8)
22. Spalletta et al. (46), Italy	Cross-sectional	103 Mild	Anosognosia; AQ-D	Depression, Apathy	NPI, specific criteria for depression and apathy in dementia.	High (8)
23. Starkstein et al. (5), Argentina, Australia, Canada	Cross-sectional	219 VM 313 Mild 169 Mod	Anosognosia; AQ-D	Apathy, Depression	HDR SCID	Moderate (6)
24. Starkstein et al. (23), Argentina, Australia, Canada	Longitudinal	213(b) 154(fu) Mild-Mod	Anosognosia; AQ-D	Depression, Apathy	SCID HAM-D AES	Moderate (7)
25. Turro-Garriga et al. (47), Spain	Prospective	177 Mild-Mod	Anosognosia; AQ-D	Depression, Apathy	NPI	Moderate (7)
26. Verhulsdonk et al. (6), Germany	Cross-sectional	12 Mild 16 Mod 1 Severe	Anosognosia; AQ-D	Depression	NOSGER-Subscale mood; GDSd NPI	High (8)
27. Vogel et al. (48), Denmark	Cross-sectional	321 Mild-Mod	Awareness; ARS, memory discrepancy rating	Depression	CSDDD	Moderate (7)

N, number of participants; NPS, neuropsychiatric symptoms; Mild-Mod, participants with mild to moderate AD; Mod, moderate AD; MCI, mild cognitive impairment; AQ-D, Anosognosia Questionnaire for Dementia; CIR, Clinical Insight Ratings Scale; GRAD, Guideline for the Rating of Awareness Deficits; ARS, Anosognosia Rating Scale; MARS, Memory Awareness Rating Scale; P-C discrepancy, patient-carer discrepancy; CDS-IU, Cognitive Difficulties Scale Index of Unawareness; PBQ, Psychobehavioural Questionnaire; SAI-E, Assessment of Insight-Extended scale; SUMD-MD, Scale for the Unawareness of Mental Disorders; RBMT, Rivermead Behavioral Memory Test; aPCRS, amended Patient Competency Rating Scale; DEX, Dysexecutive Questionnaire; NRS, Neurobehavioral Rating Scale; SCS-AD, Self-Consciousness Scale in AD; AII, Assessment of Impaired Insight; ASPIDD, Assessment Scale of Psychosocial Impact of the Diagnosis of Dementia; VSRT, Verbal Selective Reminding Test; FAI, Forgetfulness Assessment Inventory;

* *method specified in Appendix 3 (Supplementary Material); AES, Apathy Evaluation Scale; ZSRSD, Zung Self-Rating Scales for Depression; GDSd, Geriatric Depression Scale; HAM-D, Hamilton Depression Scale; HAM-A, Hamilton Anxiety Scale; MAS, Mania Assessment Scale; NOSGER, Nurse's Observational Scale for Geriatric Patients; CES-D: Center for Epidemiologic Studies Depression Scale; HRSD: Structured Interview Guide for the Hamilton Rating Scale for Depression; SCID, Structured Clinical Interview for Diagnostic and Statistical Manual of Mental Disorders; (fu), at follow up; CSDD, Cornell Scale for Depression in Dementia; BDI-II, Beck Depression Inventory-II; NPI, Neuropsychiatric Inventory; HADS, Hospital Anxiety and Depression Scale; AE, The Apathy Scale; STAI, State-Trait Anxiety Inventory; ZA, Zung Self- Rating Scales for Anxiety; BPC, Behavioral Problem Checklist*.

#### Assessment of Awareness

Among the included articles, sixteen different methods were used to assess awareness, with two studies using a battery of more than one instrument (36, 38). The Anosognosia Questionnaire for Dementia was the most commonly used instrument, reported in ten articles (5, 6, 23, 27, 28, 32, 33, 41, 46, 47). The “patient-caregiver discrepancy” approach was used by three studies (34, 35, 42) using different assessment scales. The following tools were used by two studies each: the Squire and Zouzuris Anosognosia scale (39, 43); the Anosognosia Rating Scale (ARS) (30, 48); the Assessment Scale of Psychosocial Impact of the Diagnosis of Dementia (ASPIDD) (16, 45); and the Memory Awareness Rating Scale (7, 36). The following tools were used in one study: the Guideline for the Rating of Awareness Deficits (29); and the Assessment of Impaired Insight (AII) (44). One study used sub-scores derived from insight items from a related tool (37); another study used a subtractive method (40); while Clare et al. (4) developed a multi-domain assessment for implicit and explicit awareness. The complete list and description of the instruments used by each of the included studies can be found in Appendix 2 (Supplementary Material).

#### Depression, Apathy, and Anxiety Assessment

In total, twenty-three studies addressed depressive symptoms using ten different assessment methods (4, 6, 16, 28–34, 36–48). Anxiety was assessed in four studies (4, 35, 37, 38), all of them using different measuring tools. Apathy and its relationship with awareness was assessed by eleven studies (5, 23, 27, 29, 32, 35, 37, 38, 41, 46, 47) using five different measuring tools. Appendix 3 (Supplementary Material) shows the NPS assessed, instrument used, and the number of studies that used each instrument.

#### Quality of the Studies

Within the twenty-seven studies, fifteen were ranked as high-quality (mean score = 8.33), and twelve as moderate-quality (mean score = 6.66). There were no low-quality studies (see Table 1). The overall mean score was 7.59 corresponding to moderate/high quality. Further details of quality assessment are found in Appendix 4 (Supplementary Material).

### Association Between Impaired-Awareness and Depression, Anxiety, and Apathy

The associations between impaired awareness, depressive symptoms, anxiety symptoms, and apathy are shown in Table 2. Table 3 summarizes the associations between impaired awareness and these symptoms according to quality of the studies.

**Table 2:** Results: association between impaired awareness and depression, anxiety, and apathy; data and summary of results.

**References**	**Awareness tool Awareness domain or deficit**	**NPS tool Neuropsychiatric symptom assessed**	**Statistical data**
Amanzio et al. (27)	**AQ-D** *Global awareness* *Cognitive awareness* *Behavioral awareness* *iAD awareness*	**HAMD and MAS** *Apathy* *Apathy* *Apathy* *Apathy*	β = 0.41; *p* = 0.000009^b^ β = 0.41; *p* = 0.000005^b^ β= 0.30; *p* = 0.002^b^ β = 0.41; *p* = 0.000003^b^
Bertrand et al. (28)	**AQ-D** *Awareness of condition* *Awareness of executive function*	**GDS** Depression Depression	*r* = 0.50; *p* = 0.025^a^ *r* = 0.43; *p* = 0.058^a^
Chen et al. (29)	**GRAD** *Memory deficits* *Behavioral/psychotic symptoms* *Memory deficits* *Behavioral/psychotic symptoms*	**CSDD** *Depression* *Depression* **AES-I** *Apathy* *Apathy*	*OR* = 0.74; 95% CI: 0.62–0.89; *p* = 0.001^a^ *t* = 1.10; *p* < 0.278^c^ *t* = −0.98; *p* < 0.329^c^ *OR* = 1.00; 95% CI:0.94–1.06; *p* = 0.968^c^
Cines et al. (30)	**ARS** *Memory deficits*	**GDSa** *Depression*	*t* = 6.53; *p* = 0.02^a^
Clare et al. (31)	**MARS** *Memory deficits*	**HADS** *Depression*	*r* = −0.7; *p* < 0.05)^a^
Clare et al. (4)	**Multidimensional approach+** *Implicit and explicit awareness* *Implicit and explicit awareness*	**HADS** *Anxiety* *Depression*	*F* = 3.58; *p* < 0.05^a^ *F* = 2.03; *p* = 0.138^c^
Conde-Sala et al. (32)	**AQ-D** *Global awareness* *Global awareness*	**GDS-d** *Depression* **NPI** *Apathy*	*OR* = 0.66, 95% CI: 0.54–0.82; *p* < 0.001^a^ Data not reported*^*b*^*
Conde-Sala et al. (33)	**AQ-D** *Global awareness*	**GDS-d** *Depression*	*r* = −0.46; *p* < 0.001 [mild AD group]^a^ *r* = −0.36; *p* < 0.016 [moderate AD group]^a^
Debettignies et al. (34)	**P-C discrepancy score** *Global insight*	**HAM-D** D*epression*	*r* = 0.029; *p* < 0.865^c^
Derousne et al. (35)	**P-C discrepancy score** Global unawareness Global unawareness	**PBQ** *Apathy* **ZA** *Anxiety*	*r* = 0.35. No *p*-value available^b^ *r* = −0.3. No *p*-value available^a^
Gilleen et al. (36)	**SAI** *Global* **SUMD MD** *Illness* **MARS** *Memory deficits* **PCRS** *Functioning* **DEX** *Cognitive*	**BDI** *Depression* *Depression* *Depression* *Depression* *Depression*	*r* = 0.52; *p* < 0.05^a^ *r* = −0.68; *p* < 0.001^a^ *r* = 0.68; *p* < 0.01^a^ *r* = 0.50; *p* < 0.05^a^ *r* = 0.39; *p*-not significant, not reported^c^
Horning et al. (37)	**NRS** *Insight item* *Insight item*	**NRS** *Anxiety* *Depression* **AES** *Apathy*	*F* = 4.80; *p* = 0.03^a^ *F* = 6.00; *p* = 0.02^a^ *F* = 6.64; *p* = 0.01^b^
Jacus et al. (38)	**SCS** *Global awareness* **PCRS** *Global awareness* *Global awareness*	**AES** *Apathy* **BDI-II** *Depression* **AES** Apathy **STAI-T** Anxiety	*OR* = 4.8, 95% CI: 1.14–20.8; *p* = 0.03^b^ *OR* = 4.84, 95% CI: 1.08–21.58; *p* = 0.04^a^ *OR* = 9.3, 95% CI: 2.18–39.96; *p* = 0.003^b^ ρ: 0.408; *p* = 0.009^a^
Kashiwa et al. (39)	**Squire & Zouzuris Anosognosia S**. *Global anosognosia*	**GDS** *Depression*	*r* = −0.294; *p* < 0.05^a^
Lacerda et al. (16)	**ASPIDD** *Global awareness* *Awareness of emotional state* *Awareness of social functioning and relationships*.	**CSDD** *Depression* *Depression* *Depression*	*r* = 0.27; *p* < 0.01^a^ *r* = 0.38; *p* < 0.01^a^ *r* = 0.30; *p* < 0.01^a^
Lehrner et al. (40)	**Subtracting method** *Awareness of memory deficits*	**BDI-II** *Depression*	*r* = −0.28; *p* = n.s^c^
Mak et al. (41)	**AQD** *Total* *Intellectual function* *Total* *Intellectual Function*	**AES** *Apathy* *Apathy* **GDS** *Depression* *Depression*	β = 0.41; *p* = 0.021^b^ β = 0.43; *p* = 0.015^b^ β = −0.1; *p* = 0.600^c^ β = −0.16; *p* = 0.396^c^
Nakaaki et al. (42)	**P-C discrepancy** *Awareness of memory deficits*	**CES-D** *Depression* **HRSD** *Depression*	Data missing. *P* = 0.002; *p* < 0.001^b^.
Oba et al. (43)	**Squire and Zouzuris Anosognosia** *Anosognosia for memory deficits*	**GDS** *Depression*	β = −0.25; *p* = 0.006^a^
Smith et al. (44)	**AII** *Awareness of deficits*	**GDS** *Depression*	*r* = −0.58, *p* = 0.006^a^
Sousa et al. (45)	**ASPIDD** *Awareness of disease*	**CSDD** *Depression*- *Baseline depression*- *Follow up depression*	ρ = 0.117; *p* = 0.339^c^ ρ = 0.254; *p* = 0.06^c^
Spalletta et al. (46)	**AQ-D** *Global awareness* *Global awareness*	**NPI** *Apathy* *Depression*	*t* = −3.570; *p* = 0.0005^b^ *X*^2^ = 0.1510, *p* = 0.1653^c^
Starkstein et al. (5)	**AQ-D** *Global anosognosia*	**SCID** *Apathy*	N.D^b^
Starkstein et al. (23)	**AQ-D** *Global anosognosia*	**AS** *Apathy*	*F* = 19.1; *p* = 0.0001^b^
Turro-Garriga et al. (47)	**AQ-D** *Incident global anosognosia* *Persistent global anosognosia*	**NPI** *Apathy* (incidence vs. persistence) *Apathy* (incidence vs. remission) Depression	U: 3.1 (3.6); *p* < 0.05^b^ U: 5.7 (4.2); *p* < 0.05^b^ No significant; data not available^c^
Verhulsdonk et al. (6)	**AQ-D** *Global anosognosia* *Global anosognosia*	**NPI** *Depression* **NOSGER** *Mood*	*r*: 0.53; *p* = 0.001^b^ *r*: 0.50; *p* = 0.001^b^
Vogel et al. (48)	**ARS** *Global anosognosia*	**CSDD** *Depression*	*r*: 0.07; *p* = 0.22^c^

a Negative association between impaired awareness and affective symptom (lack of awareness associated with fewer affective symptoms);

b positive association between impaired awareness and affective symptoms (lack of awareness associated with greater affective symptoms);

c *no association; between impaired awareness and affective symptom; N.D, no data available; n.s, no significant; OR, odd ratio; CI, Confidence interval; r, Pearson correlation; p, p-value; t, t-test; ρ, Spearman's rank correlation coefficient; β, standardized beta coefficient; F, F-value; X^2^, chi-square value; z, z-standardized score; γ, Goodman and Kruskal's gamma; AQ-D, Anosognosia Questionnaire for Dementia; CIR, Clinical Insight Ratings Scale; GRAD, Guideline for the Rating of Awareness Deficits; ARS, Anosognosia Rating Scale; MARS, Memory Awareness Rating Scale; P-C discrepancy, patient-carer discrepancy; PBQ, Psychobehavioural Questionnaire; SAI-E, Assessment of Insight-Extended scale; SUMD-MD, Scale for the Unawareness of Mental Disorders; aPCRS, amended Patient Competency Rating Scale; DEX, Dysexecutive Questionnaire; NRS, Neurobehavioral Rating Scale; SCS-AD, Self-Consciousness Scale in AD; AII, Assessment of Impaired Insight; ASPIDD, Assessment Scale of Psychosocial Impact of the Diagnosis of Dementia; VSRT, Verbal Selective Reminding Test; FAI, Forgetfulness Assessment Inventory; GDS, Geriatric Depression Scale; HAM-D, Hamilton Rating Scale for Depression; CSDD, Cornell Scale for Depression in Dementia; BDI-II, Beck Depression Inventory-II; NPI, Neuropsychiatric Inventory; HADS, Hospital Anxiety and Depression Scale; CAPE, Behavior Problems Checklist of the Clifton Assessment Procedures for the Elderly; SCID, Structured Clinical Interview for DSM-5; CES-D, Center for Epidemiological Studies Depression Scale; U, Mann-Whitney U Test; NOSGER, Nurse's Observational Scale for Geriatric Patients; AES, Apathy Evaluation Scale; AE, The Apathy Scale; PBQ, Psychobehavioural Questionnaire; MAS, Mania Assessment Scale; C.D, clinical diagnostic criteria; DPS, Dementia Psychosis Scale; NRS, Neurobehavioral Rating Scale; STAI, State-Trait Anxiety Inventory; ZA, Zung Self-Rating Scales for Anxiety; BPC, Behavioral Problem Checklist; DS, Disinhibition Scale; iADL, instrumental activities of daily living; [ ], subgroup analysis*.

**Table 3:** Association between impaired-awareness and depression, anxiety, and apathy.

	**Negative association^**a**^ N High Q/Mod Q**	**Positive association^**b**^ N High Q/Mod Q**	**No significant association^**c**^ N High Q/Mod Q**
Depression N: 23	13 10/3	2 1/1	8 2/6
Anxiety N: 4	4 2/2	–	–
Apathy N: 11	–	10 4/6	1 1/0

a Negative association between impaired awareness and affective symptom (lack of awareness associated with fewer affective symptoms);

b positive association between impaired awareness and affective symptoms (lack of awareness associated with greater affective symptoms);

c *no association between impaired awareness and affective symptom; N, total number of studies that assessed the association; High Q, number of high-quality studies; Mod Q, number of moderate-quality studies*.

### Impaired Awareness and Depression

Twenty-three studies assessed depression in relation to impaired awareness in mild-to-moderate AD. Among them, thirteen studies; ten of high-quality (16, 28–33, 38, 39, 43) and three of moderate-quality (36, 37, 44), found significant evidence that impaired awareness correlates with fewer depressive symptoms, with correlations ranging from 0.3 to 0.7. In other words, according to these 13 studies, having better awareness of deficits in AD correlates with more depressive symptoms.

This negative association between impaired awareness and depression was assessed by different measures and tools. For example, a high-quality study (31) reported a negative correlation between impaired awareness and depressive symptoms on the HADS (*r* = −0.7). Similarly, the study by Jacus (38), found a strong association between impaired awareness predicting less depression measured by the BDI-II (*OR* = 4.8, 95% CI: 1.0–21.5). Likewise, the moderate-quality study of Horning et al. (37) found that better insight, was related to depressed mood, as measured by the Neurobehavioural Rating Scale (*F* = 6.0).

On the other hand, two studies, one of moderate quality (42) and the other of high quality (6) found that impaired awareness was related to greater depressive symptoms (*P* = 0.002; r: 0.53, respectively).

Finally, eight studies, two high-quality (4, 45) and six moderate-quality (34, 40, 41, 46–48) concluded that there is no association, neither positive nor negative, between awareness and depressive symptoms.

### Domains of Awareness and Depression

Most of the included studies analyzed the relationship between global awareness of deficits, illness or condition, and the presence of depression or depressive symptoms. Regarding specific domains of awareness, seven studies explored *awareness of memory deficits;* most of them supporting the trend of a negative correlation between impaired awareness and depressive symptoms (29–31, 36, 43). While Nakaaki et al. (42) reported a positive association (*P* = 0.002) and Lehrner et al. (40) found no significant association (*r* = −0.28). A recent study by Bertrand et al. (28) reported a moderate positive association between having better awareness of *executive deficits* and more depressive symptoms (*r* = 0.43). Similarly, another high-quality study found that preserved awareness of *emotional-state* and preserved awareness of *difficulties of social functioning* were also correlated with greater depressive symptoms (*r* = 0.38 and *r* = 0.30, respectively) (16).

### Impaired Awareness and Anxiety

Four studies, two high-quality (4, 38) and two moderate-quality (35, 37), assessed the relationship between impaired awareness and anxiety in mild-to-moderate AD. All of them found that there was a negative association, meaning that greater impairment of awareness is associated with fewer anxiety symptoms.

Clare et al. (4) carried out a multidimensional approach with cluster analysis to identify links between degree of awareness and different variables. Regarding anxiety, they found that the group of participants with lower scores on awareness reported significantly less anxiety than those of the moderate awareness group (*F* = 3.58) (4). Jacus (38) also found that level of awareness significantly predicts the level of anxiety (*OR* = 9.3, 95% CI: 2.18–39.96). Horning et al. (37) concluded that even after controlling for impaired cognitive function, level of awareness significantly predicts level of anxiety (*F* = 4.80; and Derousne et al. (35) found a negative correlation between impaired awareness and anxiety, which persisted over time (mean follow up time 21 months, *r* = −0.3).

### Impaired Awareness and Apathy

Ten of eleven studies which assessed the relationship between impaired awareness and apathy in mild-to-moderate AD found a significant positive association, meaning that greater impairment of awareness is associated with higher levels of apathy. Of those ten studies, four were of high-quality (27, 32, 38, 46) and six of moderate-quality (5, 23, 35, 37, 41, 47). Of the high-quality studies, Amanzio et al. (27) found that apathy was a prominent feature in reduced awareness of behavioral changes (β = 0.3). One study focusing on quality of life found that impaired awareness and apathy were positively correlated among patients with mild dementia, but no significant association was seen in moderate AD (32). A study by Jacus (38) found a strong correlation between poorer awareness and greater apathy in mild-to-moderate AD, as measured by two different awareness instruments (*OR* = 4.8, 95% CI: 1.14–20.8; and *OR* = 9.3, 95% CI: 2.18–39.96). Similarly, Spalletta et al. (46) found that the severity of apathy symptoms was correlated with impaired behavioral awareness (*t* = −3.57). Likewise, six other moderate-quality studies also found this positive trend of association (5, 23, 35, 37, 41, 47). Three of these were longitudinal studies, which found that apathy had a positive correlation with impaired awareness which remained over time (*r* = 0.35) (35); that unawareness was a significant predictor of apathetic symptomatology (*F* = 19.1) (23); and that apathy was positively associated both with incidence and persistence of impaired awareness over a 12 month follow-up period (47). In contrast, one high-quality study found no significant association between apathy and any awareness domain once adjusting for dementia severity, as measured by MMSE score (*OR* = 1.00; 95% CI:0.94–1.06; *p* = 0.968) (29).

## Discussion

The overall finding of this review is that most high-quality studies suggested a negative association between impaired awareness in early AD and depression and anxiety; but a positive association between impaired awareness and apathy (see Table 3).

Although the high-quality studies support the conclusion that impaired awareness is associated with fewer depressive symptoms there were conflicting results: 13 of the 23 studies assessing this association found this negative association, while eight found no association, and two studies found a positive association. One explanation for this lack of concordance between studies might be due to the different range of assessment approaches and instruments, both for impaired awareness and depressive symptomology in people with AD. Awareness is a complex concept and cannot be considered as a unitary entity (7). Some people with dementia may be more aware of some deficits and less aware of others, which might have an impact on the association with depressive features.

Additionally, this review highlights the lack of consistency in methods of assessing impaired awareness and revealed the many different methods that are used for the same purpose in clinical research. Due to the heterogeneity in assessment tools and statistical methods used among the included studies, we did not perform a meta-analysis. As has been noted in previous literature (47, 50), not having a gold-standard method to assess impaired awareness has resulted in the development of several instruments. We found sixteen different instruments, some of them specially created for awareness in dementia, others for awareness in any disease; some assessing global-awareness, while others assessing awareness of specific domains. The variability of how awareness is theoretically understood in dementia explains why there are several methods used to assess its impairment, as has been discussed in previous reviews (7). Until a consensus of the definition of awareness and consistent measurement tools are used, or unless studies specify clearly which domain of awareness they want to address, it will be difficult to draw clear conclusions regarding impairment of awareness and its clinical correlates in AD. Similarly, the results showed that depression, anxiety and apathy in AD research are also assessed by a wide range of instruments. Moreover, cultural and gender differences in depression are also relevant. Common instruments are potentially less sensitive to adequately addressing depressive symptoms in men (51), and one study that did not find an association used an Asian sample, with the authors hypothesizing that cultural influences in minimizing depressive feelings may have confounded the results (41). In addition, some studies measured depressive symptoms with instruments which are not designed for people with dementia (34). These issues in assessing depression may partly explain the greater discrepancy in the reviewed literature for the association between awareness and depression, compared to the consistency in the results for the association with apathy and anxiety. Despite this heterogeneity in assessment tools and statistical methods used, a strength of this review is the use of quality assessments of previous studies to clarify the relationship between impaired awareness and these common NPSin AD, as the majority of high quality studies demonstrated consistent results.

The mechanisms underlying the phenomena of impaired awareness remain an area of research. Cognitive theories suggest that impaired awareness is a consequence of impairments of executive, metacognitive systems, and also due to deficits in encoding autobiographical memory related to underlying neurodegeneration in relevant brain regions (11, 52). Accordingly, neuroimaging has shown that there is an association between dysfunction of temporomedial, temporoparietal and frontal regions and the presence of unawareness in dementia (53, 54). Biopsychosocial models propose that impaired awareness might reflect the impact of the AD diagnosis (bio-component), which may generate a tension in the person with dementia, who needs to integrate the new “AD-self” with the previous “healthy-self” (psycho-component). Thus, in this model prior beliefs, expectations, and motivational factors are interrelated with awareness of disease, while the social context also plays a role in how the disease is acknowledged (social-component) (7). Understanding the biological mechanisms and possible psychosocial components of impaired awareness in dementia is important in relating deficits in awareness to the occurrence of NPS. Elucidating these mechanisms may aid clinical practice, as clarifying the psychological components of the relationship between awareness and depression or anxiety may support the use of psychosocial interventions for these common symptoms (55).

Although the association between awareness and depressive symptoms may be due in part to underlying neurodegeneration, the etiology of depression in dementia remains unclear with multiple biological and psychological mechanisms likely to be involved (20). Related to the biopsychosocial model, depression may be a reaction to the awareness of memory deficits, daily difficulties, and to the possible “anticipatory grief” of losing a previous identity. The reduction of depressive symptoms with reduced awareness may be related to both underlying biological mechanisms including neurodegeneration and to coping strategies and integration of the “new-self.” These models are not mutually exclusive and may explain different aspects of the same phenomena. Alternatively, impaired awareness may be underestimated in depressed people living with AD, and the relationship between depressive symptoms and apparently preserved awareness may be partly due to the negative bias when reporting problems (22). Depressed patients with AD might underestimate their abilities when rating themselves, and in this case, the apparent discrepancy between self-rated and informant-rated abilities, may be distorted by the depressive symptoms (43). Therefore, patients will score as apparently having more preserved awareness, but this would be due to the effect of depressive symptoms rather than an accurate assessment of illness and deficits. Moreover, at the very early stages of AD, subjective concerns about cognitive function may lead to hyper-evaluation and underestimation of performance and abilities, which may also be considered part of the continuum of distorted awareness of function in AD (56).

Apathy is defined as a disorder related to diminished motivation, interest, and expression of emotions (57). Apathy has a strong correlation with frontal lobe dysfunction, sharing this neuropathological pathway with impaired awareness (49). One study found that more severe anosognosia predicted more apathy, with two possible theories: that impaired awareness emerged as an early response to frontal lobe dysfunction, while apathy appeared with more extensive frontal lobe degeneration; or apathy may be the consequence of a poorer adaptation response to the new limitations related to dementia (23). Temporality cannot assume causality, and both theories for apathy and awareness may be correct, assuming a biopsychosocial understanding of awareness where personal factors, such as coping strategies, are involved. Moreover, most of the included studies had a cross-sectional design, thus results only reflected associations without direction among variables, therefore more longitudinal studies are needed to explore possible causalities. Finally, apathy in dementia may occur in different domains -namely cognitive, behavioral, and emotional apathy-, and the accurate measurement of apathy and its clinical relationships will depend on whether the instrument used addressed apathy as a single domain entity (i.e., the NPI) or as a multidomain syndrome (i.e., the Apathy Inventory) (58).

The biopsychosocial model might be particularly relevant to explain the association of impaired awareness, affective symptoms and apathy in early-stage AD, as it includes the relational and experiential factors as well as recognizing the impact of neurodegeneration. Understanding the underlying mechanisms in the relationship between impaired awareness and affective symptoms and apathy is essential, as treatment targets may vary if we conceptualize the phenomenon from a biological or psychological perspective. In terms of dementia care, preserved awareness or greater insight may have positive implications, as it is associated with treatment acceptance and less carer burden (10). Nonetheless, the results from this review suggest that greater awareness of deficits may be associated with a higher risk of experiencing more depressive and anxiety symptomatology. There is evidence that some specific psychological interventions are effective in managing depression and anxiety in dementia (55), however, there is currently no effective intervention for apathy (59). Although apathy and depression in dementia share some common features which often lead to misinterpretation, there is evidence that they are two different syndromes (60), requiring different therapeutic approaches. The opposite trend of association between depression and apathy in relation with awareness reported from the high-quality studies in this review, supports the distinction of depression and apathy in AD as two independent syndromes that might be explained by different underlying mechanisms. Although any explanations are speculative and likely to be multifactorial, apathy may be more biologically driven and therefore, emerges as a consequence due to neurodegeneration, while depression and anxiety might result from additional psychological factors which are more likely to occur when the person is more aware of their deficits and impairments due to dementia.

### Limitations

This review has several limitations. Firstly, we were unable to obtain raw or missing data, that may have enabled us to perform a meta-analysis and provide more conclusive evidence. Secondly, broader inclusion criteria for dementia severity and etiology could have resulted in more evidence to analyse and compare between sub-types of dementia, providing wider resources to understand different profiles of impaired awareness. Finally, we did not include gray literature and unpublished articles, which may have increased the risk of publication bias.

The most relevant limitation of the selected articles was the lack of a standard measure to assess awareness, which hindered the ability to compare results across studies. However, this is mainly due to the general limitation that emerges from the conceptualization of awareness itself, as has been previously discussed. Another limitation was the large variety of sample sizes within studies, and many studies had <100 participants; therefore, small sample sizes might have influenced the results and associations found in individual studies. Possible sources of bias and confounders were rarely addressed within studies, as for example the effect that distress or personality traits of a carer could have on rating their care-recipient performances (on the carer-patient discrepancy strategy) to assess impaired awareness. Similarly, the subjective nature of clinician judgements used in some studies could have been a source of bias that could have been avoided using more standardized standardized measuring instruments, both for impaired-awareness and for affective symptoms and apathy. It is important to note that most of the studies used the NPI to assess NPS, This is a widely used scale, however it assesses apathy and depression only as symptoms, rather than as distinct diagnoses or syndromes. In addition, as noted above, some tools used to assess depression, e.g., the HAM-D, are not specifically designed for an elderly population with dementia. This may lead to difficulties in classifying and interpreting symptoms of depression or apathy in AD, affecting the associations found with awareness in individual studies. Even though cognitive dysfunction was assessed in most studies as part of demographic characteristics or baseline measures, it was not commonly analyzed as a potential confounder in the included studies. Likewise, as was previously mentioned, gender as a moderator was not commonly assessed and no specific gender instruments were implemented. Methodology limitations were mainly related to the lack of justification of sample size and some studies had missing data. Finally, as has been highlighted in previous reviews (11), a general limitation of the methods to assess impaired awareness in AD is that the impact that awareness has on the patient's experiences, ADLs, and social relationships is usually not considered and this general measurement limitation might have affected the individual studies as well.

### Conclusion

According to the majority of high-quality studies, impaired-awareness in mild-to-moderate AD is associated with fewer depressive and fewer anxiety symptoms, but more apathy. Understanding impaired awareness is clinically important due to its high prevalence in AD and because of the consequences it has on the person with dementia, their family, and on care. Additionally, the presence of affective symptoms and apathy are challenging, and more complicated when impaired awareness of disease obstructs their management. This review provides a useful summary of the relationship between these common NPSand impaired-awareness in mild-to-moderate AD, with the methodological strength of having conducted a quality assessment of the included studies. In accordance with previous reviews (7, 11), a more accurate and standardized method to assess impaired awareness in the context of AD is needed. Understanding how awareness relates to affective symptoms and apathy is important for people with dementia and their carers, and for healthcare professionals to provide effective interventions and better integrated management of dementia, and to improve the quality of life of people living with AD and their families.

## Data Availability Statement

The original contributions presented in the study are included in the article/Supplementary Material, further inquiries can be directed to the corresponding author.

## Author Contributions

IA, GL, and JH conceived the idea for this study and interpreted the data. IA conducted the search, quality check on inclusion criteria, and drafted the manuscript and figures. JH and GL revised the manuscript. All authors contributed to the article and approved the submitted version.

## Conflict of Interest

The authors declare that the research was conducted in the absence of any commercial or financial relationships that could be construed as a potential conflict of interest.
