# Supplementary material for: The Association Between Impaired Awareness and Depression, Anxiety, and Apathy in Mild to Moderate Alzheimer's Disease: A Systematic Review
Source: Front Psychiatry. 2021 Feb 4;12:633081. doi: 10.3389/fpsyt.2021.633081 (PMC7889585; doi:10.3389/fpsyt.2021.633081)
Supplement: Supplementary file 2 [file Data_Sheet_2.PDF]

Appendix 2. Awareness assessment instruments used by each study

Instrument	Description	Number of studies
**Anosognosia Questionnaire for Dementia (AQ-D) (Starkstein et al., 2006)**	This is a 30-item questionnaire based on the patient carer discrepancy strategy; thus it must be completed by both patient and carer. It is suitable to assess impaired awareness related to mood and behavioural problems, and for cognitive deficits in AD.	**10** (Amanzio et al., 2013; Bertrand et al., 2019; Conde-Sala et al., 2012; Conde-Sala et al., 2014; Mak et al., 2015; Spalletta et al., 2012; Starkstein et al., 2006; Starkstein et al., 2010; Turro-Garriga et al., 2016; Verhulsdonk et al., 2013)
**Anosognosia Rating Scale (ARS) (Reed et al, 1993)**	The ARS is a categorical three-point scale. The clinician rates the patient's level of awareness based on cognitive testing and interviews with the patient and the caregiver. According to the clinician judgment, the patient is the classified into full, shallow, or not awareness.	**2** (Cines et al., 2015; Vogel et al., 2010)
**Guideline for the Rating of Awareness Deficits* (GRAD) (Verhey et al., 1993)**	According with the original scale, this version used a semistructured interview for patient and carers. The results were presented on a 4-level scale, that ranged between 4: "adequate awareness" to 1: "severely disturbed" awareness. Results from cognitive evaluation and the clinician observation where added in the adapted version. With this instrument the clinician was able to rate awareness of memory deficits and awareness of psychosis/behavioural problems independently.	**1** (Chen et al., 2014)
**Scale for Assessment of Insight-Extended (SAI-E) (Kemp & David, 1997)**	The extended SAI is a structured interview that explores awareness of change, awareness of difficulties due to mental conditions, and key symptoms.	**1** (Gilleen et al., 2012)
**Squire and Zouzuris Anosognosia scale* (Squire & Zouzounis, 1988)**	This adapted version consisted on a 20-item questionnaire to assess different aspects of memory impairments. Patient and carer separately rated each item using a Likert scale, comparing the patient current abilities against five years ago performance. Then, the patient-carer discrepancy strategy was used to define an anosognosia score, which ranged between -160 to +160. Higher scores represented more anosognosia for memory deficits.	**2** (Kashiwa et al., 2005; Oba et al., 2019)
**Patient-caregiver discrepancy strategy**	This strategy compares ratings given by patients regarding their performance or changes in cognition, mood, behaviour, and ADLs, against caregivers rating. Different neuropsychological tests can be used to implement this approach. The studies of this review used the following tests: The Physical Self-Maintenance Scale and the Instrumental Activities of Daily Living Scale (Debettignies et al., 1990); the Cognitive Difficulties Scale (Derousne et al., 1999); and the Short Memory Questionnaire (Naakaki et al., 2008).	**3** (DeBettignies et al., 1990; Derousne et al., 1999; Naakaki et al., 2008)

Instrument	Description	Number of studies
**Assessment of Impaired Insight (AII) (Smith et al., 2000)**	This scale used modified questions from previous instruments** to create an anosognosia questionnaire. It consists on a 22-item questionnaire to assess motor, cognitive, and affective level of awareness. Based on the patient-carer discrepancy strategy, both carer and patient rated the actual level of functioning against ten years ago. Total higher scores indicated greater anosognosia, whereas negative scores suggested exaggeration of impairments.	**1** (Smith et al., 2000)
**Neurobehavioral Rating Scale (NRS) - Item of impaired insight. (Levin et al., 1987)**	The NRS is a clinician-rated scale to assess behavioural and cognitive symptoms associated with head trauma, nonetheless, it has been found to be suitable to assess symptoms in patient with Alzheimer disease (Sultzer et al., 1995). It consisted on a 28-item questionnaire to assess particular symptoms. The NRS item that measured level of insight was used to assess overall insight.	**1** (Horning et al., 2014)
**Scale for the Unawareness of Mental Disorder scale (SUMD) - Item 1. (Amador et al., 1993)**	The item 1 from the SUMD was used to assess awareness, as it provides a single unit of global awareness of disease; scored by a Likert scale of 5 points which range between 1: “aware” to 5: “unaware”. The SUMD is a 74-item instrument to measure insight mainly used in epidemiological studies and clinical trials, yet rarely used in clinical practice due to its length.	**1** (Gilleen et al., 2012)
**Assessment Scale of Psychosocial Impact of the Diagnosis of Dementia (ASPIDD)**	This is a 30-questions scale based on the patient-carer discrepancy strategy. It was designed to assess awareness across different domains: cognitive functioning, activities of daily living, health conditions, emotional state, relationships, and social functioning. The rate of awareness range between 0 to 18, where higher scores represent less awareness.	**2** (Lacerda et al., 2017; Sousa et al., 2015)
**Memory Awareness Rating Scale (MARS) (Clare et al., 2002)**	This scale was developed to assess awareness of memory deficits. It is a 12-item memory battery, where the awareness scores are obtained by the discrepancy between the patient’s prediction of performance and actual performance. The tasks of the memory battery are mainly constituted by the Rivermead Behavioural Memory Test.	**2** (Clare et al., 2004; Gilleen et al., 2012)
**Subtractive method**	One study decided to use as measure of awareness the subtraction between two tests: The scores of delayed recalls of the Verbal Selective Reminding Test were subtracted from the Forgetfulness Assessment Inventory score.	**1** (Lehrner et al., 2015)
**Multi-domain assessment approach for explicit and implicit awareness (Clare et al., 2011)**	A multi-domain assessment was developed to asses implicit and explicit awareness. Implicit awareness was assessed with an emotional Stroop task. While explicit awareness was assessed by: metacognitive reflection about impact of the condition; memory performance monitoring; evaluative judgement of memory performance; and daily activities and socioemotional functioning.	**1** (Clare et al., 2011)

Instrument	Description	Number of studies
**The Patient Competency Rating Scale (PCRS) (Fordyce & Roueche, 1986) Amended Patient Competency Rating Scale (aPCRS)* (Bach & David, 2006)**	The PCRS is based on the patient-carer discrepancy strategy and consist of a 37-items regarding perceived performance on cognitive, emotional, and behavioural tasks. Discrepancy scores range from -148 to +148, where higher scores reflect less awareness. *The amended PCRS is the original PCRS scale with seven added items related to theory of mind.	**2** (Jacus et al., 2017; Gilleen et al., 2012*)
**Dysexecutive Questionnaire from the Behavioural Assessment of the Dysexecutive Syndrome (BADS; Wilson & Thames Valley Test Company, 1996)**	This questionnaire was designed for people with brain injury; nonetheless some questions regarding functioning are suitable for people with dementia. It consists in 20-items about functioning, including problems related with impulsivity and disinhibition. Results are obtained using the patient-carer discrepancy strategy. Discrepancy scores range from -80 to +80, where higher scores suggest less awareness.	**1** (Gilleen et al., 2012)
**Self-Consciousness Scale in AD (Gil et al., 2001)**	This is a 14-questions structured interview for the patient. It explores seven domains: identity, knowledge of cognitive disturbances, affective state, representation of the body, prospective memory, capacities for introspection, and moral judgments. The maximum score is 28, where higher scores represents better awareness.	**1** (Jacus et al., 2017)
