# Supplementary material for: The Association Between Impaired Awareness and Depression, Anxiety, and Apathy in Mild to Moderate Alzheimer's Disease: A Systematic Review
Source: Front Psychiatry. 2021 Feb 4;12:633081. doi: 10.3389/fpsyt.2021.633081 (PMC7889585; doi:10.3389/fpsyt.2021.633081)
Supplement: Supplementary file 3 [file Data_Sheet_3.PDF]

Appendix 3. Neuropsychiatric symptoms and assessment instruments

Symptom	Assessment instrument (N studies)
**Depression**	GDS (7); HAM-D (7); CSDD (5); BDI-II (3); NPI (3); HADS (2); SCID (1); CES-D (1); ZSRSD (1); NOSGER (1)
**Anxiety**	HADS (1); NRS (1); STAI (1); ZA (1)
**Apathy**	AES (4); NPI (4); AS (1); PBQ (1); HAM-D + MAS (1)

Fig 2. (N studies): Number of studies that used the instrument; GDS: Geriatric Depression Scale; HAM-D: Hamilton Depression Scale for Depression; CSDD: Cornell Scale for Depression in Dementia; BDI-II: Beck Depression Inventory-II; NPI: Neuropsychiatric Inventory; HADS: Hospital Anxiety and Depression Scale; SCID: Structured Clinical Interview for DSM-5; CES-D: Center for Epidemiological Studies Depression Scale; ZSRSD: Zung Self-Rating Scales for Depression; NOSGER: Nurse's Observational Scale for Geriatric Patients; NRS: Neurobehavioral Rating Scale; STAI: State-Trait Anxiety Inventory; ZA: Zung Self-Rating Scales for Anxiety; AES: Apathy Evaluation Scale; AS: The Apathy Scale; PBQ: Psychobehavioural Questionnaire; HAM-D+MAS: Hamilton Depression Scale for Depression + Mania Assessment Scale.
