# Supplementary material for: The Association Between Impaired Awareness and Depression, Anxiety, and Apathy in Mild to Moderate Alzheimer's Disease: A Systematic Review
Source: Front Psychiatry. 2021 Feb 4;12:633081. doi: 10.3389/fpsyt.2021.633081 (PMC7889585; doi:10.3389/fpsyt.2021.633081)
Supplement: Supplementary file 4 [file Data_Sheet_4.PDF]

Appendix 4. Quality assessment of included studies. According to the Newcastle - Ottawa Quality Assessment Scale (NOS), and the NOS adapted version for cross sectional studies.

	Selection				Comparability		Outcome/ Exposure		Total Score	Quality
**Study**	**Sample Rep.**	**Sample Size**	**Non-resp.**	**Tool (**)**	**Main Factors**	**Addit. Factors /Conf**	**Assessment (**)**	**Stats/ Folow-up**	**range: 1-10**	
Amanzio, 2013	*	X	*	**	*	*	**	*	9	High
Bertrand, 2019	*	x	x	**	*	*	**	*	8	High
Chen, 2014	*	X	*	**	*	*	**	*	9	High
Cines, 2015	*	X	*	**	*	*	**	*	9	High
Clare, 2004.	*	X	*	**	*	X	**	*	8	High
Clare, 2011	*	X	*	*	*	*	**	*	8	High
Conde-Sala, 2012	*	X	X	**	*	*	**	*	8	High
Conde-Sala, 2014.	*	X	*	**	*	*	**	*	9	High
DeBettignies, 1990	*	X	X	*	*	*	*	*	6	Mod
Derousne, 1999	*	X	X	*	*	*	*	*	6	Mod
Gilleen, 2012	*	X	X	**	*	X	**	*	7	Mod
Horning, 2014	*	X	X	**	*	X	**	*	7	Mod
Jacus, 2016	*	X	X	**	*	*	**	*	8	High
Kashiwa, 2005	*	X	X	**	*	*	**	*	8	High
Lacerda, 2017	*	X	*	**	*	*	**	*	9	High
Lehrner, 2015	*	X	X	*	*	*	**	*	7	Mod
Mak, 2015	*	X	X	**	*	X	**	*	7	Mod
Nakaaki, 2008	*	X	X	*	*	X	**	*	6	Mod
Oba, 2018	*	X	X	**	*	*	**	*	8	High
Smith, 2000	*	X	X	*	*	*	**	*	7	Mod
Sousa, 2015	*	X	X	**	*	*	**	*	8	High
Spalletta, 2012	*	X	X	**		*	**	*	8	High
Starkstein, 2006	*	X	X	**	*	X	**	X	6	Mod
Turro-Garriga,2016	*	X	X	**	*	X	**	*	7	Mod
Verhulsdonk, 2013	*	X	X	**	*	*	**	*	8	High
Vogel et al., 2010	*	X	X	**	*	X	**	*	7	Mod

Low quality: <5; Moderate quality: 5 to 7; High quality: 8 to 10. Sample Rep.: Representativeness of the sample; Non-resp: Non-respondents; Addit: additional factors; Conf: confounders
